# Biomimetic Growth of Hydroxyapatite on Electrospun CA/PVP Core–Shell Nanofiber Membranes

**DOI:** 10.3390/polym10091032

**Published:** 2018-09-17

**Authors:** Jiazi Hou, Yihuan Wang, Hailong Xue, Yanli Dou

**Affiliations:** Key laboratory of Automobile Materials of Ministry of Education, College of Materials Science and Engineering, Jilin University, Changchun 130025, China; houjiazi@jlu.edu.cn (J.H.); yihuan18@mails.jlu.edu.cn (Y.W.); xuehl14@mails.jlu.edu.cn (H.X.)

**Keywords:** electrospinning, core–shell, polyvinylpyrrolidone, cellulose acetate, hydroxyapatite

## Abstract

In this study, cellulose acetate (CA)/polyvinylpyrrolidone (PVP) core–shell nanofibers were successfully fabricated by electrospinning their homogeneous blending solution. Uniform and cylindrical nanofibers were obtained when the PVP content increased from 0 to 2 wt %. Because of the concentration gradient associated with the solvent volatilization, the composite fibers flattened when the PVP increased to 5 wt %. Fourier transform infrared spectroscopy (FTIR) and X-ray diffraction (XRD) results confirmed the existence of a hydrogen bond between the CA and PVP molecules, which enhanced the thermodynamic properties of the CA/PVP nanofibers, as shown by differential scanning calorimetry (DSC) and thermogravimetric analysis (TGA) results. To analyze the interior structure of the CA/PVP fibers, the water-soluble PVP was selectively removed by immersing the fiber membranes in deionized water. Scanning electron microscopy (SEM) and transmission electron microscopy (TEM) indicated that the PVP component, which has a low surface tension, was driven to the exterior of the fiber to form a discontinuous phase, whereas the high-content CA component inclined to form the internal continuous phase, thereby generating a core–shell structure. After the water-treatment, the CA/PVP composite fibers provided more favorable conditions for mineral crystal deposition and growth. Energy-dispersive spectroscopy (EDS) and FTIR proved that the crystal was hydroxyapatite (HAP) and that the calcium to phosphorus ratio was 1.47, which was close to the theoretical value of 1.67 in HAP. Such nanofiber membranes could be potentially applicable in bone tissue engineering.

## 1. Introduction

Electrospinning technique has gained considerable attention because it can generate ultrafine fibers in the micrometer to nanometer scale by applying high electric fields [1,2,3]. During electrospinning, viscoelastic polymer solutions are elongated in the electric field and solidified on the collector to form nonwoven nanofiber membranes [2,4]. Given their unique properties, such as porosity and large surface area, electrospun nanofibers have been widely utilized as excellent candidates for scaffolds in tissue engineering, carriers in drug delivery, matrixes in wound dressing, and so on [5,6,7,8].

As an important bone tissue filling material, hydroxyapatite (HAP) has been applied for artificial bone replacement in tissue engineering, mainly because of its composition similarity with the inorganic component in natural bone tissues [9]. In addition, hydroxyapatite has been used as a suitable environment for cell seeding and nutrient diffusion for the healthy growth of osteoblasts. Realizing a porous structure is a common strategy for this purpose. To date, several technologies, including combined salt leaching, microsphere sintering, phase separation, and rapid prototyping, have been used to generate porous scaffolds in the in-depth study of tissue engineering scaffolds [10]. Given that the web-like structure precisely simulates the topology of the extracellular matrix, the electrospinning membranes are a promising selection and could provide a favorable environment for the growth of new tissues. Therefore, blending electrospinning and biomimetic mineralization can typically achieve a porous nanofiber scaffold as a composite material of HAP to mimic the natural bone. Sheikh et al. [11] introduced electrospun polyurethane nanofibers containing HAP inorganic nanoparticles in their research. The results confirmed good dispersion of HAP nanoparticles on the well-aligned nanofibers. Jin et al. [12] combined a modified electrospinning method with biomineralization to produce fluffy and porous poly-*l*-lactic acid nanofibers with HAP. Researchers recently produced an electrospun poly(ε-caprolactone)/HAP composite mat with excellent physical and chemical properties that could be used as a promising substitute for bone regeneration [13,14]. Besides, poly(lactic acid–*co*–glycolic acid) [15], polyvinyl alcohol [16], and some natural materials [17,18] have been studied as well. All of these polymers and their composite blend not only mimic the natural tissues structure but are also compatible for HAP nucleation and growth. However, the above polymers are all hydrophobic. Hydrophilicity is a key factor that promotes the cell affinity of scaffolds and extends their applications in tissue engineering [12,19]. Therefore, a hydrophilic and biocompatible scaffold that can provide nucleation sites to form a mineral crystal must be fabricated.

Cellulose acetate (CA) electrospun fibers are superior in terms of stability, biocompatibility, and hydrophilicity [20]. As a derivative of cellulose, CA can be easily dissolved in most organic solvents and electrospun to form fibers, suggesting that CA has a significant advantage in electrospinning as an ideal substitute for natural cellulose [21]. However, the lack of chemical affinity and low oxidizability of CA fibers prevent their widespread applications. To satisfy more demands, the performance of cellulose acetate could be enhanced by blending it with appropriate additives. Kendouli and Castillo-Ortega reported on CA-based electrospun nanofibers [22,23], but the selection of hydrophilic additives and the application in tissue engineering requires further investigation. Polyvinylpyrrolidone (PVP), which is a polymer produced by the polymerization of *N*-vinyl-2-pyrrolidone monomers, presents the characteristics of moisture absorption, excellent solubility, and biocompatibility [24]. It is widely used in many industries, such as pharmaceuticals, paints, adhesives, and biological engineering materials [25,26]. To further develop the promising functional cellulose acetate fibers and exploit its application in tissue engineering, we selected PVP to modify cellulose acetate fibers by homogenous electrospinning.

In this paper, CA/PVP homogeneous electrospinning was performed, and core–shell nanofibers were observed because of the phase separation and low surface tension of PVP. Scanning electron microscopy (SEM) and transmission electron microscopy (TEM) were employed to investigate the surface morphology and interior structure of the fibers. The conformation, crystallinity, and thermal properties were analyzed by Fourier transform infrared spectroscopy (FTIR), X-ray diffraction (XRD), and differential scanning calorimetry (DSC) and thermogravimetric analysis (TGA), respectively. Prior to the biomimetic mineralization, the fibrous membranes were immersed in deionized water and subsequently in ethanol solution. The obtained cellulose scaffold with mineralized HAP to imitate the component of native bone was produced by simulated body fluid (SBF) immersion method. The composition of the mineral crystal was further investigated by FTIR and energy-dispersive spectroscopy (EDS) to demonstrate its potential application for tissue engineering.

## 2. Materials and Methods

### 2.1. Materials

CA (*M*_n_ = 50,000, 39.7 wt % acetyl content) and PVP (*M*_n_ = 58,000) were purchased from Aladdin Chemistry Co. Ltd. (Shanghai, China). Acetone and ethanol were obtained from Tianjin Xintong Chemical Agent Company (Tianjin, China). Sodium chloride (NaCl), sodium hydrogen carbonate (NaHCO_3_), sodium sulfate (Na_2_SO_4_), sodium hydroxide (NaOH), potassium chloride (KCl), calcium chloride (CaCl_2_), potassium phosphate dibasic trihydrate (K_2_HPO_4_·3H_2_O) and 1.0 mol/L hydrochloric acid (HCl) were procured from Beijing Chemical Works (Beijing, China). Magnesium chloride hexahydrate (MgCl_2_·6H_2_O) was procured from Xilong Chemical Co. Ltd. (Guangdong, China). Tris (hydroxymethyl) aminomethane (Tris) was obtained from Aladdin Industrial Corporation. Deionized water was made using a water purification system (Smart Q-15, Shanghai Hitech Instrument Co., Ltd. (Shanghai, China) in the laboratory.

### 2.2. Electrospinning

The CA solution was prepared by dissolving 10% (*w*/*w*) CA in the *co*-solvent of water/acetone (15/85, *w*/*w*) and stirring for 3h. Varying PVP contents (0%, 0.5%, 1%, 2%, and 5%, *w*/*w*) were added to the CA solution. As the precursor solutions, they were fully stirred for 12 h to obtain transparent and homogeneous solutions.

A 5 mL syringe containing the precursor solution was controlled by a programmable pump (America Harvard Apparatus Co., Ltd., Holliston, MA, USA) at a constant feed rate of 1.5 mL/h. A 5 cm × 5 cm aluminum foil was used to collect the as-spun fibers. A high voltage supply (Tianjin Dongwen High Voltage Facility, Tianjin, China) that generated a voltage of 20 kV was employed between the syringe nozzle and the aluminum foil with a distance of 15 cm. Electrospinning was conducted at a humidity of 40%–50% and a room temperature of 22–25 °C.

### 2.3. Mineralization in the SBF Solution

The electrospun fibers were immersed in deionized water (approximately 20 mL) and shaken gently for 24 h to remove the PVP. Prior to mineralization, the treated fibrous membranes were dried in vacuum at 40 °C.

The water-treated CA and CA/5% PVP membranes were separately immersed in breakers containing 30 mL of ethanol solution (20 wt %) and shaken for 5 min to facilitate a through infiltration. Then, the membranes were gently washed thrice with SBF to remove the ethanol completely. Afterward, 20 mL of fresh SBF were filled to the breakers, and the SBF was changed every two days. The breakers were incubated at 37 °C to obtain mineralized-state scaffolds. After incubations for 3, 6, and 9 days, the SBF was removed, and deionized water was filled several times to exclude the inorganic ions. The preparation produced for the SBF solution used for the biomineralization has been discussed in the literature [12,27].

### 2.4. Measurement and Characterization

#### 2.4.1. Solution Properties

The conductivity, viscosity, and surface tension of the precursor solution were measured using a JYW surface tension instrument (Chengde Youte Detection Instrument Co., Ltd., Chengde, China), DDS-11A conductivity meter (Shanghai Shengci Instrument Co., Ltd., Shanghai, China) and NDJ-1 rotary viscosimeter (Shanghai Yutong Instrument and Equipment Factory, Shanghai, China), respectively. To avoid accidental errors, all samples were tested at least thrice.

#### 2.4.2. SEM and EDS

The electrospun fibers were observed by SEM (JSM-6700F, JEOL, Ltd., Tokyo, Japan) at 8 kV to observe the surface morphology of the samples. In view of the poor conductivity, all samples were coated with over 4-nm-thick gold prior to SEM. The elemental analysis of the incubated mineral was investigated by an EDS system attached to the SEM.

#### 2.4.3. TEM

The fiber membranes were observed by TEM at 100 kV (H-600, Hitachi, Tokyo, Japan). The copper mesh was laid on the aluminum foil for approximately 5 s to obtain the test samples during the electrospinning prior to TEM observation.

#### 2.4.4. FTIR

FTIR spectroscopy (Tensor 27, Bruker, Karlsruhe, Germany) was applied to the analysis the participating fibers’ chemical structure. All test samples were prepared with spectroscopic-grade KBr at a hydraulic pressure of 400 kg. The FTIR spectra were obtained under absorption mode and covered from 600 to 4000 cm^−1^.

#### 2.4.5. XRD

To analyze sample composition and crystalline state, the XRD spectra profiles of CA and CA/PVP fibers were determined by an X-ray diffractometer (D/Max2500/PC Rigaku, Tokyo, Japan) with CuK_α_ (λ = 1.54056 Å) at a 50 kV voltage and a 30 mA current. The scanning range was 5° to 60°, and the scanning speed was 5°/min.

#### 2.4.6. Thermal Analysis

Each sample was measured using DSC (Q200, TA, New Castle, DE, USA) and TGA (Perkin–Elmer Pyris, Perkin–Elmer, Waltham, MA, USA) to evaluate thermal properties. The DSC samples (approximately 3–5 mg) were heated ranging from 20–300 °C under a 50 mL/min nitrogen flow, whereas the TGA samples (approximately 10 mg) were heated from 50 to 800 °C at a heating rate of 10 °C/min.

## 3. Results and Discussion

### 3.1. The Properties of Spinning Solutions

The physical parameters, such as conductivity, shear viscosity, and surface tension, critically influenced the fiber morphology. The data of the solution properties are displayed in Table 1. It could be seen that the conductivity decreased gradually with increasing concentration of PVP. The main reason for this is was that the PVP limited the solution conductivity [28,29]. Meanwhile, with the introduction of PVP, the viscosity of the precursor solutions obviously increased. The increase in viscosity was ascribed to the sufficient molecular entanglement generated between the PVP and CA molecules, which enhanced the intermolecular force. Moreover, the surface tension of the precursor slightly decreased, mainly because of the addition of PVP. The surface tensions of varying PVP contents ranging from 0.5% to 5% PVP were 30.05, 30.08, 30.17, and 31.11 mN/m, which were lower than that of CA. Therefore, PVP was likely to be distributed outside of the fiber during the electrospinning. This hypothesis will be proved in a subsequent discussion.

### 3.2. Morphology of the CA/PVP Nanofibers Membranes

**Figure 1:**
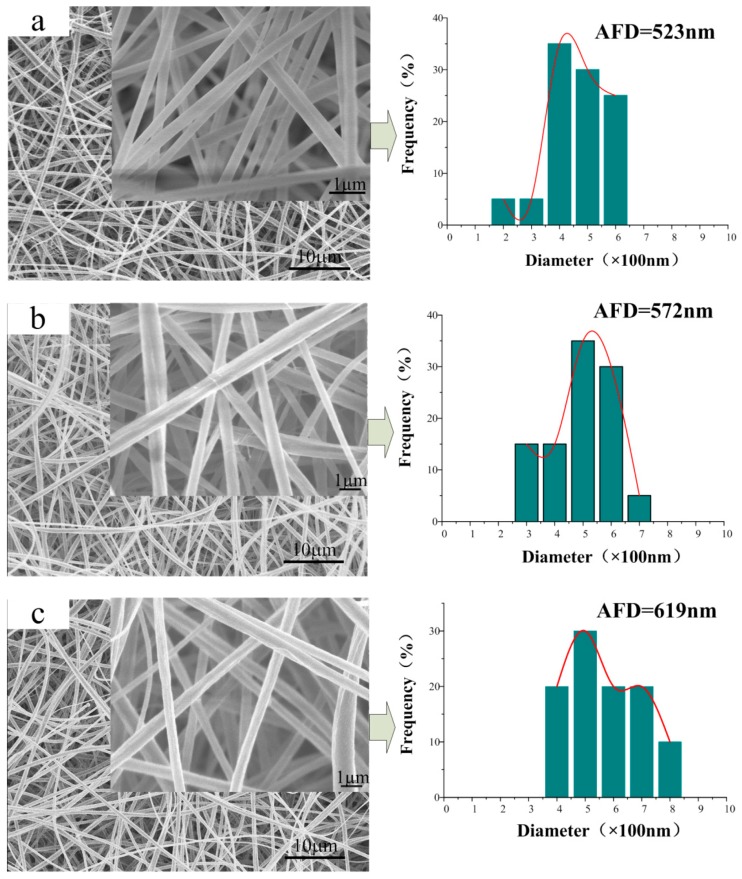

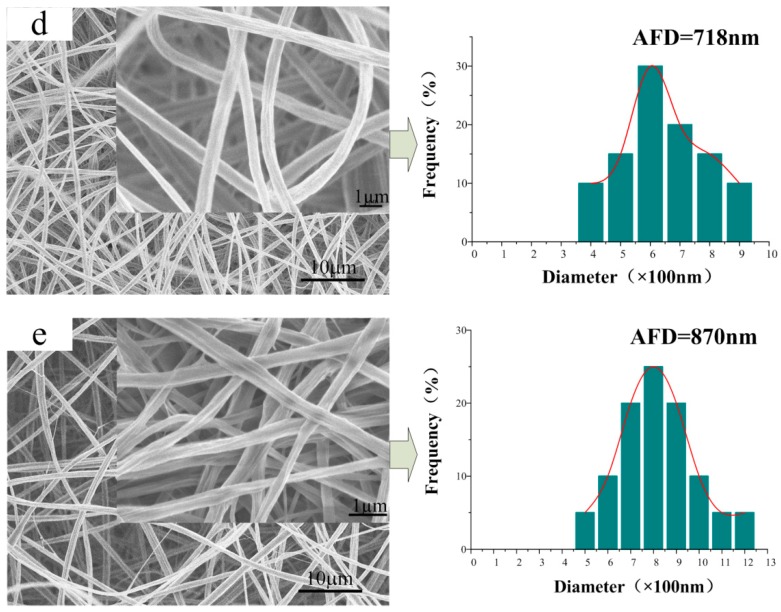
SEM images of a series of CA/PVP nanofibers with varying PVP contents (**a**) 0%; (**b**) 0.5%; (**c**) 1%; (**d**) 2%; (**e**) 5%. The pictures on the right correspond to the diameter distributions.

Figure 1 shows the SEM images and diameter distributions of the CA/PVP fibers with varying contents of PVP. Smooth, uniform, and cylindrical nanofibers were produced from pure CA. As the PVP proportion increased, the continuous and uniform morphology of the fibers was maintained. However, it groove-embedded and flat nanofibers were more likely to be obtained; the cylindrical structure disappeared in the case for CA/5% PVP fibers. The rough surface of the hybrid fibers might be due to the evaporation of the solvent. The jet instability, which was due to the increased PVP content, was the reason that solvent- and polymer-rich areas were more easily generated on the thermodynamically unstable fiber surface. With the rapid volatilization of the solvent, many grooves emerged on the surface layer. In addition, the formation of flat fibers could be attributed to the concentration gradient associated with the solvent volatilization. The volatilization rate of acetone on the surface was greater than the diffusion rate of the internal acetone, which trigged the final solidification of the jet with a flat cross section. For fibers with a higher PVP proportion, the chain entanglement limited the solvent volatilization inside the fibers.

Another factor affected by the PVP component was the diameter of the obtained nanofiber morphology. The average fiber diameter (AFD) of pure CA was 523 nm, whereas that of hybrid nanofibers increased from 572 to 870 nm. Under the synergistic effect of high viscosity and low conductivity, the viscous resistance of the adequate polymer content and the reduced electrostatic traction force increased the fiber diameter. Moreover, the diameter distribution was also widened with increased PVP concentration because of the unstable jet resulting from the addition of more PVP.

### 3.3. Composition of the CA/PVP Hybrid Nanofibers

**Figure 2:**
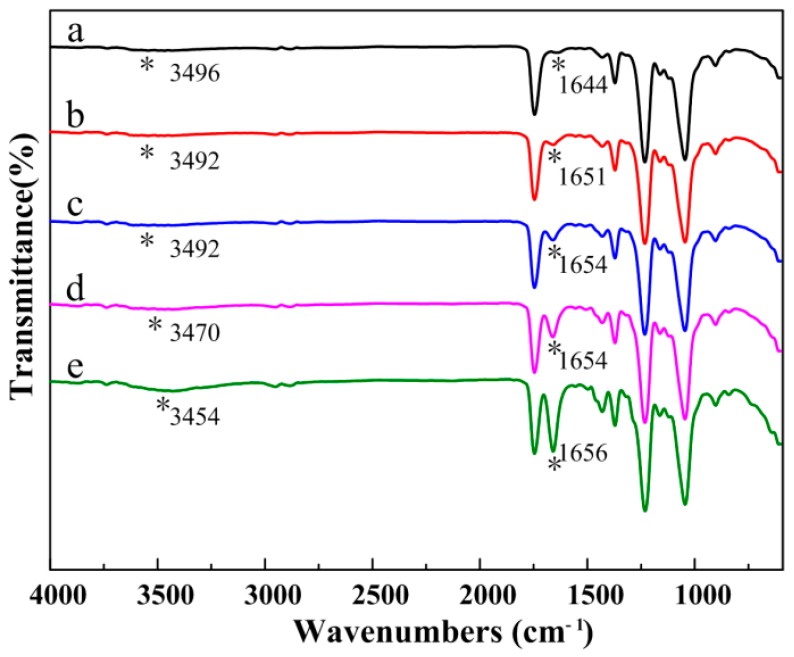
FTIR spectra of the CA/PVP hybrid nanofibers with varying PVP quantities (**a**) 0%; (**b**) 0.5%; (**c**) 1%; (**d**) 2%; (**e**) 5%. *: the position of corresponding characteristic peaks.

Figure 2 depicts the FTIR spectra of the CA/PVP hybrid nanofibers. Similar curves were obtained before and after the introduction of PVP, demonstrating that no chemical changes occurred in the process of blending. The main absorption peaks of pure CA were represented by the stretching vibrations of C=O from ester groups (1743 cm^−1^), C–O from the carboxylic group (1232 and 1045 cm^−1^), the O–H group (3496 cm^−1^), and the bending vibrations of the CH_3_ deformation for the acetate substituent groups (1371 cm^−1^) [30,31,32]. The infrared spectra of the CA/PVP hybrid nanofibers displayed peaks for the stretching vibration of the tertiary C–N group at 1295 cm^−1^ and the bending vibration of the amide carbonyl group at 1651 cm^−1^, which were the characteristic peaks of PVP [33]. As the PVP content increased, it could be observed that the band at 3492 cm^−1^ became more significant with the shift to lower wavenumbers, whereas the peak at 1651 cm^−1^ shifted to higher wave numbers, and the peak intensity increased. The findings suggested that the carbonyl group of PVP (acting as proton acceptors) and the hydroxyl group in CA (acting as proton donors) inclined to form hydrogen bonds.

**Figure 3:**
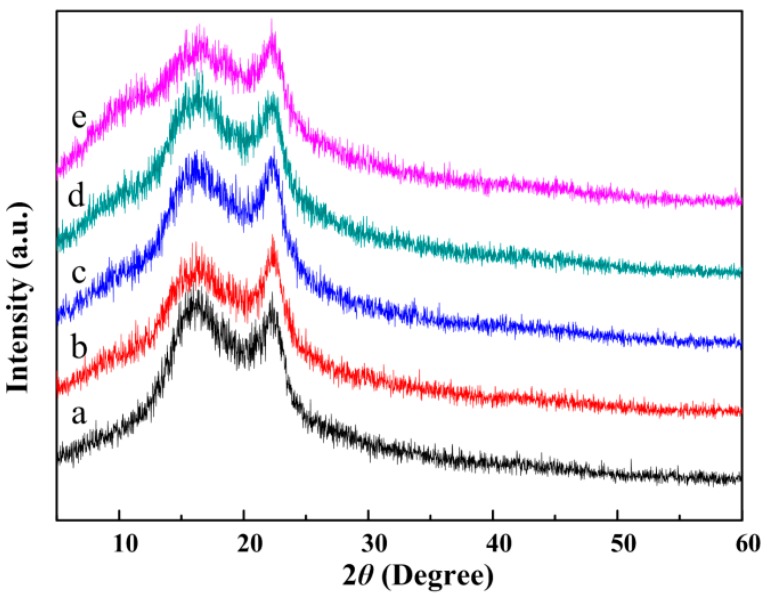
XRD patterns of the CA/PVP hybrid nanofibers with varying PVP quantities (**a**) 0%; (**b**) 0.5%; (**c**) 1%; (**d**) 2%; (**e**) 5%.

Figure 3 presents the XRD patterns of pure CA and CA/PVP hybrid nanofibers. The pure CA nanofibers showed two broad peaks at Bragg angles 2θ = 16.0° and 22.3°, which were ascribed to the semicrystalline phase of the cellulose acetate [34,35]. With the introduction of PVP, no well-defined sharp peak was attributable to PVP, although a broad band was achieved at 13.4°, thereby suggesting that the PVP had an amorphous nature [36]. As the PVP loading increased, the characteristic peak intensity of CA substantially decreased. The reason for this phenomenon is that the CA and PVP chains were blended at the molecular level. Moreover, the formation of hydrogen bonds led to the disordered packing of CA chains.

### 3.4. Thermal Properties

**Figure 4:**
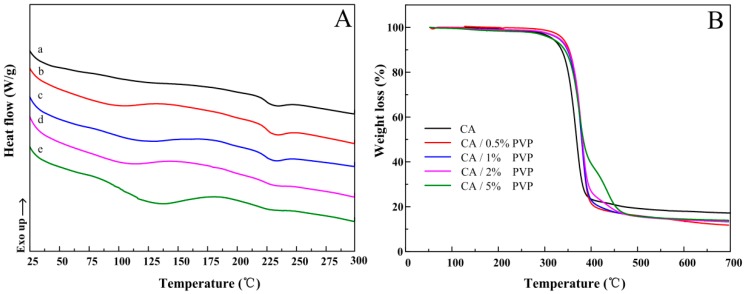
(**A**) DSC and (**B**) TGA curves of the CA/PVP hybrid nanofibers with varying PVP quantities: (**a**) 0%; (**b**) 0.5%; (**c**) 1%; (**d**)2%; and (**e**)5%.

The thermal properties of CA/PVP hybrid nanofibers were measured by DSC and TGA. The corresponding data are listed in Table 2. As shown in Figure 4A, the CA nanofibers showed a broad endothermic peak located at 231.89 °C, which was the melting temperature of CA. For the CA/PVP as-spun fibers, the melting peaks shifted to a lower temperature, and the curves had new broad endotherms, which were located between 80 and 180 °C because of PVP dehydration. By increasing the PVP amount to 0.5 wt % in the CA fibers, the melting temperature was slightly decreased to 229.62 °C and the melting peak became less prominent. These findings showed that the ordered association of the CA molecules was reduced due to the presence of PVP component, demonstrating that the CA molecular chains were highly constrained by the amorphous PVP.

Figure 4B presents the thermal decomposition expressed in terms of weight loss as a function of temperature for the electrospun fibers. The weight loss before 300 °C was ascribed to the loss of the absorbed moisture and/or the evaporation of the trapped solvent. A single decomposition step from 300 to 400 °C was observed for the pure CA membrane. In the case of hybrid fibers, two degradation steps were observed. The first weight loss mainly corresponded to the degradation of CA, which had a decomposition temperature of approximately 370 °C. In the second weight loss process, the polymer residues were further degraded at approximately 430 °C because of the decomposition of the PVP content [37]. As shown in Figure 4B, the continuous addition of PVP in the hybrid fiber increased the initial and decomposition temperatures, implying that the thermal stability of the hybrid materials was improved. The enhanced stability at higher temperatures resulted from the stronger interaction between the polymers due to the formation of hydrogen bonds.

### 3.5. Microstructure of the CA/PVP Hybrid Nanofibers

**Figure 5:**
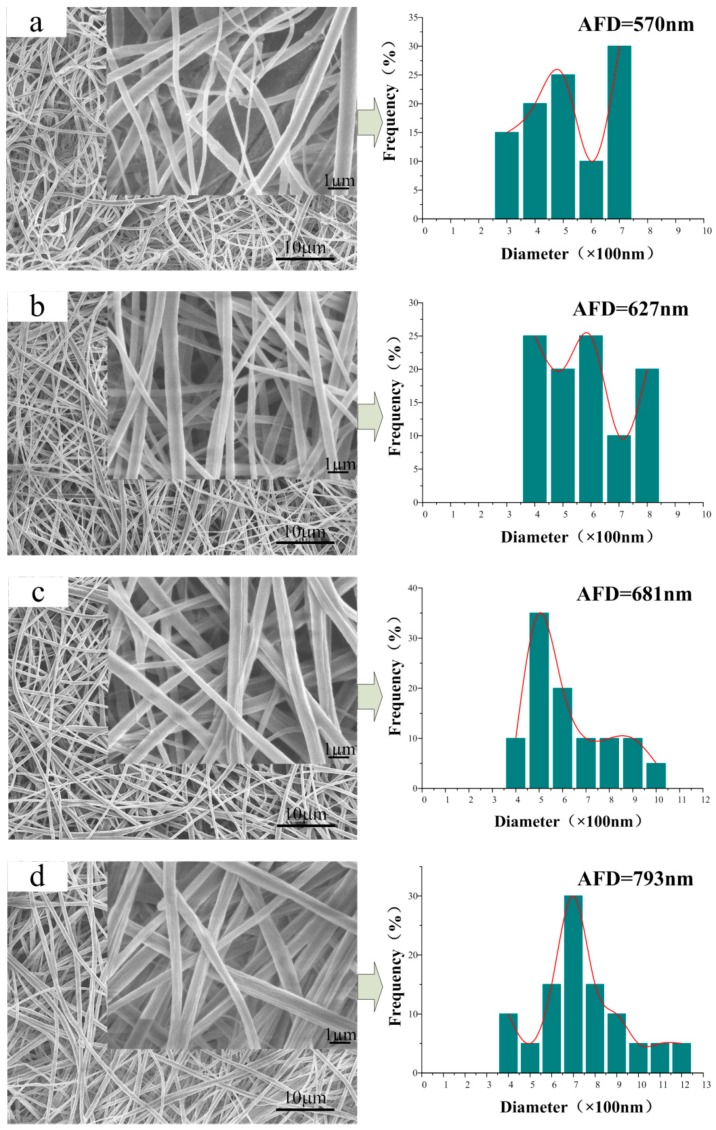

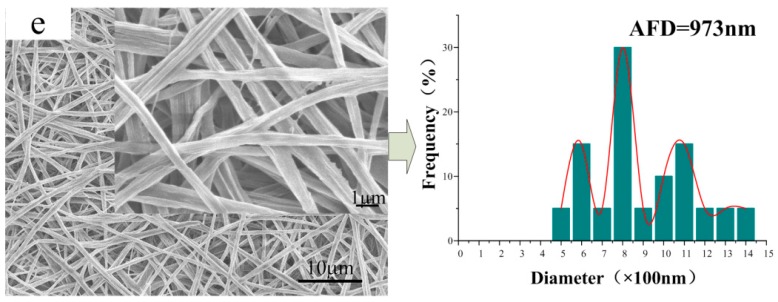
SEM images of a series of water-treated CA/PVP hybrid fibers with varying PVP contents: (**a**) 0%; (**b**) 0.5%; (**c**) 1%; (**d**) 2%; (**e**) 5%. The pictures on the right correspond to the diameter distributions.

Figure 5 shows the SEM images of CA/PVP nanofibers with varying PVP contents after water treatment. Given that PVP is water-soluble, its presence could be estimated by comparing the fiber morphology before and after the treatment on which basis the fiber microstructure could be further deduced. As shown in Figure 5a, the treated CA fibers remained in cylindrical form, and the average fiber diameter increased slightly (from 523 to 570 nm; swelling rate 8.98%). For the CA/PVP hybrid fibers, the surface was no longer smooth, and a number of cavities and grooves appeared. As the PVP content increased, the fiber diameters exhibited an increasing trend, and the swelling rates were 9.62%, 10.01%, 10.44%, and 11.8% for CA/0.5%PVP, CA/1%PVP, CA/PVP2%, and CA/5%PVP, respectively. The appearance of longitudinal grooves and cavities could be ascribed to the phase separation and the removal of the discontinuous-phase PVP gathering in the fiber. The main reason for the increased swelling ratio was the removal of PVP and the outward migration of insoluble CA component, which thereby increased the swelling ratio.

**Figure 6:**
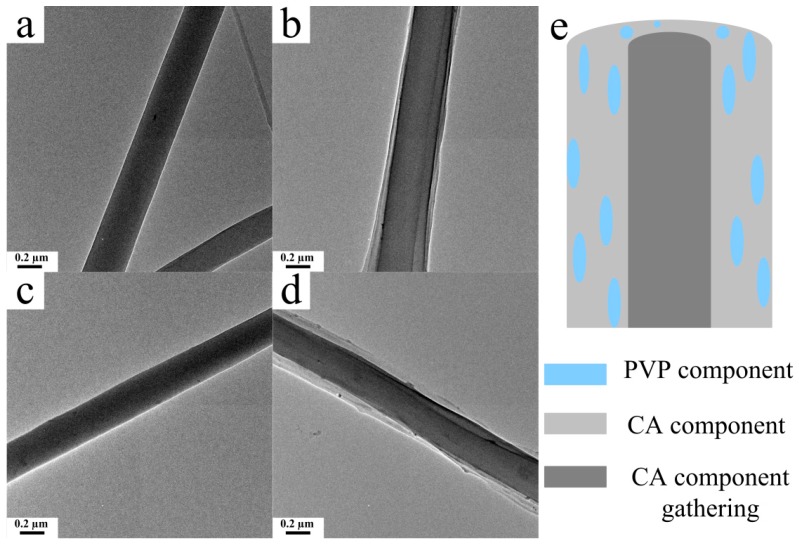
TEM images of the pure CA fiber (**a**) before and (**c**) after the water treatment. The CA/5% PVP fibers (**b**) before and (**d**) after the water treatment. (**e**) Schematic diagram of the CA/PVP hybrid fiber.

The interior distributions of the CA/PVP hybrid nanofibers were displayed by the TEM images (Figure 6). No significant change occurred between the water-treated CA fiber and the untreated fiber. However, as shown in Figure 6b, an obvious core–shell structure was achieved by electrospinning CA and PVP homogenous precursor with a single capillary [38,39]. The core–shell structure with a sharp interface was retained after water treatment. The reason for the generation of core–shell nanofibers could be as follows: The PVP component was driven toward the fiber exterior during the electrospinning process because of the attraction of the low surface energy, which was the key factor in forming the core–shell structure [40,41]. The high fraction of acetone allowed for the high mobility of the CA polymer chains, which caused a large-scale continuous phase of the CA component to form the core structure, thereby generating a phase separation. The schematic diagram of the CA/PVP hybrid fiber is shown in Figure 6e.

### 3.6. Mineralization of the Nanofibers

**Figure 7:**
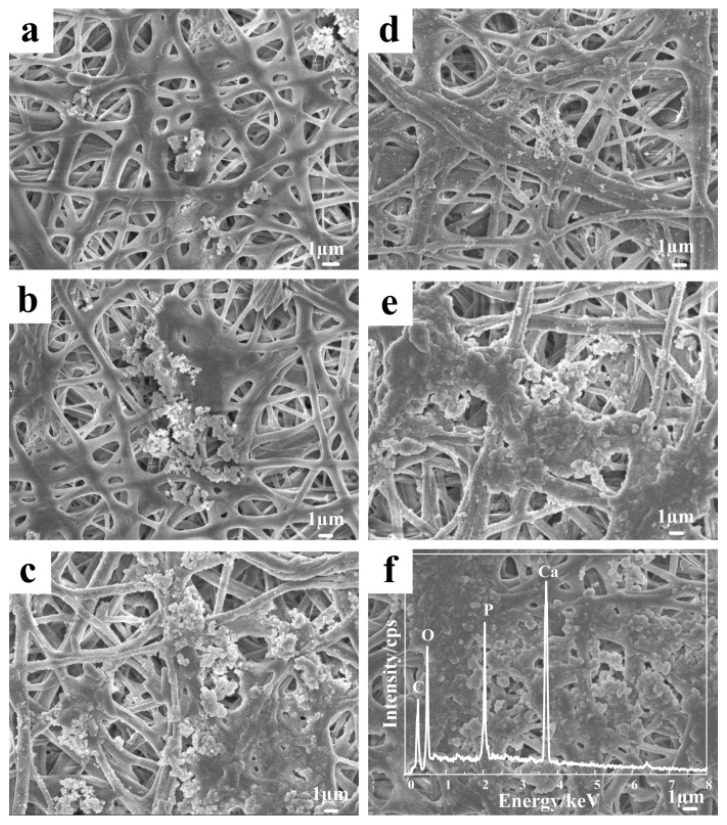
SEM images of the water-treated CA nanofibers after incubation for various days (**a**) 3 days, (**b**) 6 days, and (**c**) 9 days. Water-treated CA/5% PVP fibers after incubation for various days (**d**) 3 day, (**e**) 6 days, and (**f**) 9 days with EDS spectrum.

As shown in Figure 7, the SEM images confirmed the production of mineral crystals on the fiber surface after mineralization for a number of days. Figure 7a shows that very few mineral crystals were found on the CA fibers surface on the third day. On the sixth and ninth days, the crystals increased in number and assembled to form a larger mineral phase. For the CA/PVP fibers, crystals appeared and even existed in the gap between fibers on the third day. The amount of mineral crystals increased rapidly with prolonged incubation time, whereby a continuous mineral layer was formed on the cellulose nanofibers after nine days of incubation. After water treatment, CA/5% PVP fibers provided more favorable conditions, such as addition grooves and cavities, for the crystal deposition and growth. This would be beneficial for bone tissue applications.

The nature of the mineral phase was studied by EDS. The spectra revealed that the main elements constituting the incubated mineral were carbon, oxygen, calcium, and phosphorus. Calcium and phosphorus could have originated only from the mineral phase, suggesting that the mineral deposited on the surface of the cellulose fibers might have been similar to hydroxyapatite, which has the molecular formula Ca_10_(PO_4_)_6_(OH)_2_. Furthermore, the calcium to phosphorus (Ca/P) ratio was 1.47, which was close to the theoretical value of 1.67 in HAP [42], thereby illustrating that these crystals were still calcium-deficient. If the samples were to be applied to the human body as a bone scaffold, calcium content must be improved.

**Figure 8:**
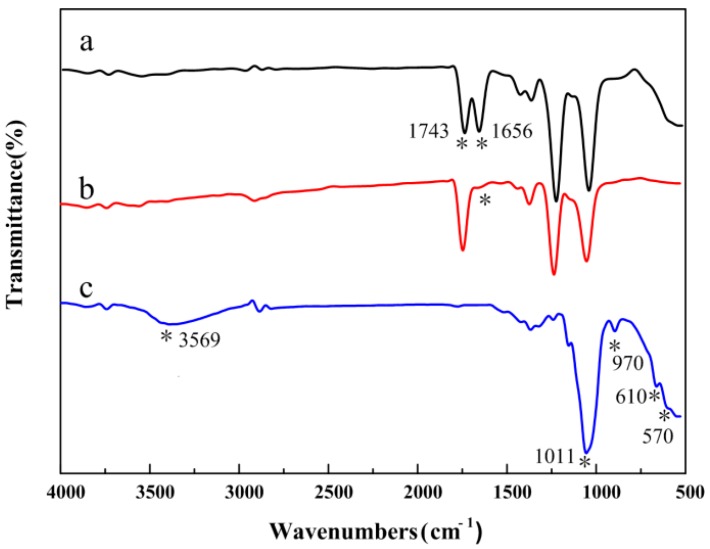
FTIR spectra of (**a**) CA/5% PVP fibers; (**b**) water-treated CA/5% PVP fibers; and (**c**) incubated CA/5% PVP fibers.

Figure 8 depicts the FTIR spectra of the electrospun, water-treated, and incubated CA/5% PVP fibers. For the water-treated CA/5% PVP fibers, the characteristic peaks of the amide carbonyl group at 1656 cm^−1^ were not observed, suggesting that PVP was removed successfully after the water treatment. The incubated CA/5% PVP spectrum confirmed that the disappearance of the characteristic peak at 1743 cm^−1^ was due to the stretching vibration of carbonyl groups, indicating that the cellulose acetate was converted into cellulose. Additionally, the peaks at 1011 and 970 cm^−1^ were the absorption peaks of the stretching vibration of phosphate and the peaks at 610 and 570 cm^−1^ were ascribed to the vibration of phosphate [43,44]. The peak at 3569 cm^−1^ could be assigned to the hydroxyl group of HAP, which further confirmed that HAP existed in the fibers.

## 4. Conclusions

CA/PVP hybrid nanofibers were prepared by electrospinning. The average diameter of the nanofibers varied in the range of 500–900 nm. The effect of various parameters was emphasized in this study, such as fiber morphology, thermal behavior, interior structure, and mineralization growth of HAP on the fiber. With increasing PVP content in the hybrid fiber, the uniform and cylindrical morphology gradually disappeared, and flat fibers with many grooves were obtained when the PVP content was 5 wt %. The concentration gradient and the solvent volatilization deteriorated the morphology. The FTIR and XRD curves indicated that the carbonyl group in PVP and the hydroxyl group in CA inclined to form hydrogen bonds, resulting in the disordered packing of CA chains. Additionally, the thermal stability of the hybrid fibers was enhanced because of the formation of hydrogen bonds between the two polymers. To remove the PVP component, the fiber membranes were treated with deionized water. After the water treatment, the fibers lost their original shape, and many grooves and cavities appeared. The swelling ratios of the hybrid fibers were 8.98%, 9.62%, 10.01%, 10.44%, and 11.8% for CA/0%PVP, CA/0.5%PVP, CA/1%PVP, CA/2%PVP, and CA/5%PVP, respectively. The TEM results showed that the CA/5% PVP fibers had a core–shell structure, which was retained after the water treatment. In view of the immiscibility of CA and PVP polymers, the PVP—with a low surface tension—was driven toward the shell section to form a discontinuous phase, whereas the high-content CA increased to form the core section, thereby generating a phase separation. Thus, the CA/PVP core–shell fibers were produced by homogenous electrospinning without requiring a coaxial needle. The water-treated fiber membrane was immersed in ethanol solution and then biomineralized in SBF to obtain the biomimetic crystalline HAP layer. The CA/PVP fibers could provide more favorable conditions for mineral crystal nucleation and growth. FTIR and EDS analyses proved that the crystal on the fiber surface was HAP and that CA was converted into cellulose. Moreover, the Ca/P ratio of the mineral crystal was 1.47, which was close to its theoretical value of 1.67 in HAP. The high HAP induction bioactivity of the as-spun cellulose nanofibers made them applicable in the biomimetic fabrication of fiber/HAP composites. Such composites may be promising bone tissue engineering materials.

## Figures and Tables

**Table 1:** The properties of electrospun cellulose acetate (CA)/polyvinylpyrrolidone (PVP) solutions.

Sample	CA Content (%)	PVP Content (%)	Acetone/H_2_O Mass (g)	Conductivity (mS/cm)	Shear Viscosity (mPa·s)	Surface Tension (mN/m)
CA	10	0	25.5/4.5	24.4	270	34.20
CA/0.5% PVP	10	0.5	25.5/4.5	23.6	311	34.10
CA/1% PVP	10	1	25.5/4.5	22.0	321	33.93
CA/2% PVP	10	2	25.5/4.5	16.3	335	33.76
CA/5% PVP	10	5	25.5/4.5	11.5	363	33.36

**Table 2:** TGA curves of CA/PVP hybrid nanofibers analysis results *.

Sample	*T*_m_ (°C)	*T*_g_ (°C)	*T*_i_ (°C)	*T*_d1_ (°C)	*T*_d2_ (°C)
CA	231.89	188.28	267.43	365.21	-
CA/0.5% PVP	231.84	189.31	267.12	378.35	428.27
CA/1% PVP	231.79	193.37	269.68	378.45	434.22
CA/2% PVP	231.58	207.31	269.54	379.36	434.26
CA/5% PVP	229.62	207.75	270.85	375.38	434.20

* *T*_m_: the melting temperature; *T*_g_: the glass transition temperature; *T*_i_: the initial temperature of decomposition; *T*_d1_: the degradation temperature of CA; T_d2_: the degradation temperature of PVP.
